# Suppression of Extinction with TMS in Humans: From Healthy Controls to Patients

**DOI:** 10.1155/2006/393924

**Published:** 2006-11-27

**Authors:** Massimiliano Oliveri, Carlo Caltagirone

**Affiliations:** ^1^Dipartimento di PsicologiaUniversità di PalermoItaly; ^2^Fondazione "Santa Lucia" IRCCSRomaItaly; ^3^Clinica NeurologicaUniversità di Roma Tor VergataItaly

## Abstract

We review a series of studies exemplifying some applications of single-pulse and paired-transcranial magnetic stimulation (TMS) in the study of spatial attention and of its deficits. We will focus primarily on sensory extinction, the failure to consciously perceive a contralesional sensory stimulus only during bilateral stimulation of homologous surfaces. TMS studies in healthy controls show that it is possible either to interfere or modulate the excitability of the parietal cortex during sensory (i.e. tactile and visual) attentional tasks, thus reproducing a condition of virtual extinction. TMS studies in patients with unilateral (mainly right) brain damage show that the modulation of the unbalance in cortical excitability between the two cerebral hemispheres transiently improves contralesional sensory extinction. These studies show the possible application of TMS not only as a research method in healthy subjects, but also as a tool for inducing brain excitability changes in patients with sensory extinction, which could be useful for supporting the rehabilitation of this deficit.

## 1. Introduction

The power of TMS to transiently disrupt and modulate the neural activity in focal brain regions [1–3] makes it a technique of choice to look deeper into the physiological bases of conscious sensory perception than it was possible with evoked potential measurements alone. A useful way to explore the neural mechanisms underlying conscious experience is to study patients in whom, as a consequence of brain damage, conscious and unconscious mental processes can be dissociated [4]. An example of such dissociation is sensory extinction, a pathological condition following unilateral (usually right) brain damage in which a left sensory stimulus fails to access consciousness only when a right stimulus is presented simultaneously [5,6]. Therefore, left sensory extinction offers a model for studying perceptual awareness from its transitory absence. Extinction has been interpreted as a pathological bias in spatial attention, favoring the ipsilesional side of space as a result of the over control of behavior by the intact but disinhibited hemisphere of brain-damaged patients [7, 8].

We will review a series of studies, which analyzed the performance on sensory discrimination tasks of both healthy subjects and patients with contralesional sensory extinction. These studies exemplify three of the major potential contributions of TMS in this field: the transient disruption of focal cortical activity in healthy controls to establish the causal role and the timing of the contribution of a given cortical region in a behavior; the application of TMS to the study of functional brain connectivity; the application of TMS to human patients to examine the compensatory cortical plasticity that occurs in response to a lesion.

## 2. Contralateral extinction induced by single-pulse and repetitive TMS in healthy controls

A first group of studies used TMS as a "virtual lesion" method [3] to transiently disrupt the cortical ac-

ISSN 0953-4180/06/$17.00 © 2006 – IOS Press and the authors. All rights reserved tivity of parietal and frontal cortices in healthy subjects, mimicking the effects of specific neurological lesions temporarily. The first demonstration of attentional, "extinction-like", effects of TMS was provided by Pascual-Leone et al. [9], applying repetitive TMS (rTMS) trains at 25 Hz frequencies over parietal, occipital and temporal cortices during a visual attentional task. The main results showed that rTMS to the right and left parietal cortices induced selective extinction of visual stimuli presented to the side contralateral to TMS during simultaneous stimulation of both visual fields. By contrast, rTMS of the occipital cortex interfered with detection of contralateral visual stimuli both in single and bilateral presentation modalities.

Similar findings have been obtained in the tactile domain. A study by our group provides an example of how TMS can provide chronometric information about the timing of activation of parietal and frontal cortices in tactile attention tasks [10]. We applied single-pulse TMS over left/right parietal (along the intraparietal sulcus) and frontal (along the inferior frontal sulcus) scalp sites of healthy subjects receiving unimanual or bimanual tactile stimulations. TMS of the right parietal cortex interfered with the detection not only of contralateral, but also of ipsilateral stimuli. These effects were mainly evident during bimanual stimulation, thus reproducing a contralateral or ipsilateral tactile extinction. Regarding the timing of the effects, we found that the contribution of the right parietal and frontal cortices was critical between 20 and 40 ms after tactile stimulation, a finding reproducing the results obtained by previous investigators [11–13] with unimanual stimulation.

Seyal et al. [14] tested the effects of right parietal TMS on ipsilateral tactile perception in a group of healthy subjects carrying out a tactile detection task with the right thumb. Single-pulse TMS delivered 50 ms prior to the delivery of the electrical stimulus increased the sensitivity to cutaneous stimulation, compared to baseline or frontal TMS trials. These findings were interpreted as the result of a TMS-induced transient disruption of the ipsilateral parietal cortex, resulting in disinhibition of the contralateral (i.e. left) parietal cortex during the sensory discrimination task, probably mediated by transcallosal effects. This study not only sheds light on the cortical circuitry essential for spatial attention (in which lesions are associated with extinction and neglect) but also stresses the important role of interhemispheric interactions in the distribution of attention to tactile space, providing the basis for a TMS-induced suppression of contralesional extinction in patients with unilateral brain damage.

## 3. Modulation of sensory perception by paired-TMS of the parietal cortex

Paired-TMS protocols can offer a way to modulate the excitability of a specific cortical region, inducing either inhibition or facilitation depending on the interval between the two magnetic stimuli (ISI). In the motor cortex, the test motor potential evoked in the intrinsic hand muscles is inhibited by a preceding conditioning subthreshold stimulus (CS) at short ISIs of between 1 and 5 ms (intracortical inhibition, ICI), while with longer ISIs of 8–15 ms the test responses are facilitated (intracortical facilitation, ICF) [15]. The inhibition is likely to reflect the activity of GABAergic interneurons, while facilitation would depend on the activation of intracortical fibers by the CS, inducing local release of glutamate.

Recent studies suggest that this technique could be applied to modulate the excitability of cortical regions even outside the primary motor cortex, in order to study the neural mechanisms of sensory perception in healthy subjects as well as of sensory extinction in brain-damaged patients. Oliveri et al. [16] used a paired-TMS protocol on the right parietal cortex of a group of healthy subjects to investigate the presence of a pattern of excitatory/inhibitory interactions during tactile perceptual tasks. Fifteen healthy volunteers received threshold electrical stimuli to the left thumb. Electrical stimuli were followed after delays of 10–40 ms by paired-TMS pulses, with a CS (70% of motor threshold) followed at various ISIs (ISI: 1, 3, 5, 10, 15 ms) by a suprathreshold (130% of motor threshold) stimulus (TS). Paired-TMS of the parietal cortex could selectively modulate the perception of contralateral tactile stimuli, compared with single-pulse TMS, depending on the ISI between the CS and the TS: paired-stimuli disrupted tactile detection (as compared with single-pulse TMS alone) when delivered 1 ms apart; in contrast, during paired-TMS with ISI of 5 ms, detection of the tactile stimuli approached the baseline detection rate with no TMS.

In a recent study [17] we replicated these findings in the visual domain. When applied at an interval of 150 ms from the presentation of threshold unilateral or bilateral visual dots, paired-TMS of the right parietal cortex with ISI of 3 ms interfered with contralateral visual perception compared with single-pulse TMS; on the other hand, paired-TMS with ISI of 5 ms facilitated contralateral visual perception compared with single-pulse TMS.

These findings argue for the existence of a supramodal pattern of excitatory and inhibitory interactions in the right posterior parietal cortex during spatial attentional tasks. Future studies could investigate the presence of specific alterations of these interactions in patients with sensory attentional disorders. We will address this topic in the following section of the manuscript.

## 4. TMS modulation of sensory extinction in brain-damaged patients

Among the multiple deficits of perception and exploratory behavior that constitute the neglect syndrome, extinction of contralesional sensory stimuli is a cardinal sign indicating an attentional disorder [4–6]. In fact, extinction, like neglect [6] has been interpreted as the result of a pathological attentional bias toward the right space, due to the disinhibition of the healthy (i.e. left) hemisphere following the release of the reciprocal inhibition from the affected one [7,8]. From this hypothesis comes the idea to modulate contralesional extinction by transiently disrupting the activity of the unaffected hemisphere of brain-damaged-patients using TMS.

Oliveri et al. [18] applied single-pulse TMS to frontal, parietal and prefrontal scalp sites of the unaffected hemisphere of right-brain-damaged (RBD) and left-brain-damaged (LBD) patients, after an interval of 40 ms from unimanual or bimanual tactile stimulation. Suprathreshold TMS of the left frontal cortex significantly reduced contralesional extinction rates during bimanual stimulation in RBD group, while it was ineffective during unimanual stimulation. The extinction improvement was not limited to patients with neglect or extinction but was also evident in those showing only contralateral somatosensory deficits. No TMS effects were encountered in the LBD group.

This work represents the first example of the application of TMS in human patients to improve a sensory/cognitive function. As in the animal models of neglect [19], transient disruption of one hemisphere is able to restore the distribution of attention to the contralesional side of space, thereby improving extinction. These findings support a model of hemispheric imbalance, with a disinhibition of the healthy hemisphere, as the physiological basis of the right hyperattention in the neglect syndrome. Moreover, the findings extend previous observations about hemispheric asymmetries in the processing of somatosensory stimuli. It is also worth noting that only contralateral extinctions, primarily produced by deficits at the attentional level, were influenced by left hemisphere TMS, whereas the perception of contralesional unimanual stimuli was not influenced. This finding suggests that the contralateral deficits ameliorated by TMS-disruption of the healthy hemisphere have an attentional component.

In another study [20] the same authors tested the differential effects on contralesional extinction of single-pulse vs. paired-TMS with 1 and 10 ms ISIs delivered over the left parietal and frontal scalp sites of a group of eight RBD patients with tactile extinction. The aim of this study was to combine the interfering mechanism of action of single-pulse TMS with distinct facilitatory/inhibitory effects on task performance due to the modulatory action of the preceding conditioning stimulus. According to the model postulating a relative disinhibition of the unaffected hemisphere in extinction patients, left paired-TMS pulses with ISIs that potentiate inhibitory cortical interneurons should transiently improve left tactile extinctions; on the other hand, paired-TMS pulses with ISIs that potentiate (or do not inhibit) cortical excitability should worsen, or leave unchanged, left extinctions. Another aim of the study was to analyze the chronometry of the spatial distribution of tactile attention in frontal and parietal regions. This question was addressed by tracing the effects of TMS of the parietal and frontal areas on left extinctions at various intervals (10, 20, 30, 40 ms) after tactile stimulation. The CS intensity was 70% of motor threshold, while the TS intensity was 130% of motor threshold.

Single-pulse TMS improved the extinction rate. Paired-TMS had distinct effects on contralesional extinction depending on the ISI and on the time of application with respect to the tactile stimulus: paired-TMS with ISI of 1 ms improved extinction rate significantly more than single-pulse TMS; paired-TMS with ISI of 10 ms did not affect left extinction rate, with a complete reversal of the effects of single-pulse TMS. TMS effects were different in the parietal vs. frontal cortex depending on the time of TMS application: the effect on parietal cortex appeared significantly earlier (20–30 ms after tactile stimulation) than the effect on the frontal cortex, reaching its maximum 40 ms after tactile stimulation, as already demonstrated in the previous study [18].

These results provide an example of how TMS can be useful to test the chronometry of spatial attention, thanks to its time-resolution that is beyond the power of neuroimaging studies. Moreover, the findings suggest the possibility to activate intracortical inhibitory and excitatory circuits even outside of the motor cortex, which could be clinically beneficial in patients with neurological disorders involving sensory perception.

In order to test the potential therapeutic effects of paired TMS, we have tested in a paired-TMS study [21] the hypothesis that the pattern of inhibitory/excitatory interactions sub-serving right tactile perception is dynamically altered in the unaffected (left) parietal cortex of RBD extinction patients: we expected that the relative hyper-activation of the left hemisphere would be selectively evident during bimanual stimulation, due to the imbalanced inter-hemispheric competition for attention resources that is selectively present in this stimulation condition. We have analyzed the effects of paired-TMS pulses, delivered at 1 ms ISI over the left parietal cortex, on right (i.e. ipsilesional) tactile detection, comparing the patients' performance with that of a control group of healthy subjects. The critical comparison was between the TMS-effects on right tactile detection during unimanual right (a non-extinction condition) vs. bimanual (when contralesional extinction emerged) stimulation tasks.

The paradigm of paired-TMS consisted of a subthreshold CS followed by a suprathreshold TS at 1 ms ISI. The CS intensity was 70% of motor threshold, while the TS intensity was 130% of motor threshold. The TS was delivered at 30 ms delay following the onset of the tactile stimulus.

The main findings show that single-pulse TMS to the left parietal cortex similarly disrupts right tactile perception in both patients and controls in either unimanual or bimanual detection tasks. The effects of paired-TMS with 1 ms ISI on right tactile perception are different in RBD patients vs. controls depending on the stimulation condition (i.e. unimanual vs. bimanual). In the control subjects paired-TMS disrupts right tactile perception more than single-pulse TMS during both unimanual and bimanual stimulation, as previously demonstrated [16]; in RBD patients, the interfering effect of paired-TMS on right perception is not present during bimanual stimulation, and is even reversed in sign. On the other hand, when patients are tested with unimanual right stimulation, paired-TMS to the left parietal cortex has the same disrupting effect as in normal subjects. In addition, the results confirm previous findings [18,20], showing that both single-pulse and paired-TMS of the left (undamaged) hemisphere ameliorate left tactile extinctions of RBD patients, the effect of paired-TMS with 1 ms ISI being significantly greater than that of single-pulse TMS.

The basic mechanism of disruption of contralateral tactile perception by single-pulse TMS seems to be intact in RBD patients during both unimanual and bimanual stimulation tasks. What is altered in the left parietal cortex of RBD patients is the interaction between conditioning and test magnetic pulses which, by analogy with the motor cortex, probably reflects the excitability of intrinsic interneuronal GABAergic and glutamatergic circuits. However, it is worth noting that any inference of cortical excitation and inhibition starting from behavioral data is speculative at present. Data from the motor cortex suggest that inhibition and facilitation are generated by cortical elements, such as cortico-cortical connections, oriented parallel to the surface of the brain. The activation of such fibers by CS during paired-TMS induces release of glutamate, which activates both excitatory and inhibitory interneurons, leading to local increases in cerebral blood flow (CBF), as detected with neuroimaging recordings. Consistent with these data, a recent study using Positron Emission Tomography showed an increase of CBF with paired-TMS at both inhibitory and excitatory ISIs between CS and TS [22]. By extension, one can speculate that a reduced intracortical inhibition is likely to determine a reduced metabolic activation, as studied with functional magnetic resonance imaging (fMRI), in the corresponding cerebral regions. In other words, the lack of inhibitory effects of paired TMS in the left parietal cortex of RBD patients during bimanual stimulation and the reduced activity of the left hemisphere observed with fMRI or event-related potentials during extinction, as compared with non-extinction, conditions [4,23,24] may both reflect the reduction of intracortical inhibition to the left side of the brain.

## 5. Conclusion

We have reviewed a number of studies, using single-pulse and paired-TMS and different experimental paradigms, which show how this technique can be of fundamental importance in elucidating the mechanisms of spatial attention and of its deficits (i.e. sensory extinction). Some of these studies open the way to the possible application of TMS not only as a research method, but also as a tool for inducing long-lasting brain excitability changes, which could be useful for the treatment of various neurological disorders.

## Acknowledgements

We wish to thank the invaluable contribution to this work of P. Cicinelli, G. Koch, M.M. Filippi, P. Pasqualetti, P. M. Rossini, S. Torriero, R. Traversa.
